# Investigation of Sesamol on Myeloperoxidase and Colon Morphology in Acetic Acid-Induced Inflammatory Bowel Disorder in Albino Rats

**DOI:** 10.1155/2014/802701

**Published:** 2014-01-30

**Authors:** Phani Krishna Kondamudi, Hemalatha Kovelamudi, Geetha Mathew, Pawan G. Nayak, Mallikarjuna C. Rao, Rekha R. Shenoy

**Affiliations:** Department of Pharmacology, Manipal College of Pharmaceutical Sciences, Manipal University, Manipal, Karnataka 576104, India

## Abstract

*Background*. Inflammatory bowel disease (IBD) is a chronic inflammatory disorder of gastrointestinal tract of immune, genetic, and environmental origin. In the present study, we examined the effects of sesamol (SES), which is the active constituent of sesame oil in the acetic acid (AA) induced model for IBD in rats. *Methods*. The groups were divided into normal control, AA control, SES, and sulfasalazine (SS). On day 7, the rats were killed, colon was removed, and the macroscopic, biochemical, and histopathological evaluations were performed. *Results*. The levels of MPO, TBARS, and tissue nitrite increased significantly (*P* < 0.05) in the AA group whereas they reduced significantly in the SES and SS treated groups. Serum nitrite levels were found to be insignificant between the different groups. *Conclusions*. The mucosal protective effects of sesamol in IBD are due to its potential to reduce the myeloperoxidase and nitrite content.

## 1. Introduction

Inflammatory bowel disease (IBD) with its two main forms: Crohn's disease (CD) and ulcerative colitis (UC), usually affects the quality of life of the patients. The pathological findings include loss of mucosal integrity and inflammatory cell infiltration [1]. The pathophysiology remains elusive [2–11]. Apart from these, reactive oxygen species (ROS) also play an important role in the mucosal damage and proved to play an important role in the progression of the disease [12, 13]. In acute colitis patients, along with epithelial cell damage, there is marked infiltration of inflammatory cells. The damaged epithelial cells are rapidly repaired by restitution, proliferation, and differentiation. The most critical process for mucosal healing is restitution [14, 15]. The drug therapy is limited to immunity and microbes instigated IBD and has a lot of limitations due to their toxicities [16, 17]. Moreover, some drugs like NSAIDs, isotretinoin, antibiotics, oral contraceptives, mycophenolate mofetil, ipilimumab, rituximab, and sodium phosphate act as causative agents for inflammatory bowel like diseases [18]. Nowadays, the use of medicinal plants or their active ingredients have been increased in the treatment of IBD to those which are unresponsive to the standard therapy. In particular, the focus has been shifted to the relatively nontoxic food additives but lack of sufficient scientific understanding related to their mechanism of action limits their use into the main stream of medical care. The present drug, sesamol (SES), is a component of traditional health food in various Asian countries [19]. It protects against atherosclerosis, hypertension, and aging [20, 21]. It has also been explored for wound healing [22], antioxidant [23], anti-inflammatory [24], and free radical scavenging activity [25]. The anti-inflammatory effect of SES on dinitrochlorobenzene (DNCB) model of colitis has been studied [26]. In acute models like trinitrobenzene sulphonic acid (TNBS) induced colitis also, the activity of sesame oil has been explored [27] but not the active constituent sesamol. So, the present study investigates the anti-inflammatory effect of sesamol in an acute model of colitis. Of several models for the induction of IBD, we have used acetic acid because intrarectal administration of dilute acetic acid into rodents or rabbits leads to epithelial injury and increased permeability followed by an acute mucosal/transmural inflammation in a dose-dependent manner [28]. The mimicking features which this model shows while correlating with human IBD are vasopermeability, prolonged neutrophils infiltration, and increased production of inflammatory mediators [29]. So, the present study involves the anti-inflammatory activity along with mucosal healing of SES in acetic acid (AA) induced IBD.

## 2. Methods

### 2.1. Chemicals

Acetic acid, 2-thiobarbituric acid, and trichloro-acetic acid wrere obtained from (HiMedia Laboratories, Mumbai, India). Sesamol, O-dianisidine dihydrochloride, and Griess reagent were obtained from Sigma-Aldrich, St. Louis, MO, USA. Total protein kit was obtained from Thermo Fisher Scientific Inc., Rockford, IL USA. Analytical grade chemicals were used.

### 2.2. Animals

Healthy inbred female albino rats of Wistar strain (160–200 g) were used. The rats were kept in air-conditioned room maintained at a temperature of 23 ± 2°C with a 24 hr light-dark cycle. The animals had free access to standard pellet diet and water *ad libitum*. The experiments were approved by Institutional Animal Ethical Committee (IAEC) (vide no. IAEC/KMC/88/2011-2012) and were carried according to the guidelines of the Committee for the Purpose of Control and Supervision of Experiments on Animals (CPCSEA), Government of India. Intrarectal administration of AA was carried out under ketamine anesthesia.

### 2.3. Induction of Colitis and Treatments

The protocol and dosing strategies were taken from the previous study with a slight modification [30]. 24 rats were divided into 4 groups of 6 animals each as follows: normal control, AA control, Sesamol treated (SES), and sulfasalazine treated (SS). From day 1 to day 3, SES and SS treated groups were given 100 mg/kg *p.o.* whereas AA treated group was given 0.3% CMC. On day 4, all the animals except control group were given acetic acid (3% v/v) by intracolonical administration, 2 h after administration of the drugs to the animals in their respective groups. Volume of acetic acid administered was 2 mL of 3% (v/v) by intracolonical route. After anesthetizing the rats with ketamine, an intravenous cannula (21G) was inserted into the rectum [6-7 cm from the anus]. Animals were kept in that position for a few minutes and later washed with saline to remove the remaining acetic acid solution. (Make sure that the instilled acetic acid should not come out as it causes writhing activity in the rectum.) The control animals were instilled with distilled water. On day 6, drug administration was continued to the respective groups and finally on the 7th day all the animals were sacrificed. The entire colon starting from caecum was taken and placed on a slab for measuring the length and weight. Around 6-7 cm of proximal part of colon was taken for biochemical estimation which includes nitrite, TBARS, and MPO by placing them in physiological buffer pH 7.4 until the homogenization of the samples was carried out. A small part of proximal colon was taken for histopathological study and stored in 10% formalin until the histological studies were carried out. Before killing the animals, blood was collected, serum and plasma were separated individually from each rat, and the samples were estimated for nitrite levels.

### 2.4. Homogenization of Samples

The samples were homogenized in an ice container at a concentration of 10% (w/v) in 11.5 g/L solution of potassium chloride by using a glass homogenizer. After this, the homogenized samples were centrifuged at 10,000 rpm for 15 min at 4°C. The supernatant was pipetted out with a microtiter pipette and separated into aliquots for individual biochemical estimations.

### 2.5. Assay of Colonic MPO

Myeloperoxidase (MPO) is an enzyme found in the intracellular granules of neutrophils which can be utilized as an indirect measure of the neutrophil content of the tissue sample [31]. The entire estimation was carried out in a 96-well plate and the readings were taken on a microplate reader (ELx800, BioTek Instruments, Inc., Winooski, VT, USA) at 490 nm. 50 *μ*L of sample was taken in duplicate. To this, 250 *μ*L of ODA-H_2_O_2_ was added which comprises of 680.45 mg of potassium dihydrogen orthophosphate in 100 mL of distilled water and the pH was adjusted to 6.0. ODA solution includes 0.167 mg of ODA in 1 mL of phosphate buffer of pH 6.0. Finally, ODA-H_2_O_2_ was prepared by adding 1 mL of 30% of H_2_O_2_ in 1 mL of ODA solution. After addition, the reading was noted at 5 min and 15 min. After this, 4 M H_2_SO_4_ was added to stop the reaction and once again the reading was noted. The concentrations of MPO at subsequent time intervals were determined from standard plot which uses horse radish peroxidase as standard. Note that H_2_O_2_ and ODA solutions are light sensitive, so they were wrapped in aluminum foil. The entire experiment was done under dark conditions especially addition of ODA-H_2_O_2_ solution.

### 2.6. Assay of Lipid Peroxides in Colonic Homogenates

Malondialdehyde (MDA) which was formed by the breakdown of polyunsaturated fatty acids (PUFA) serves as an index for determining the extent of peroxidation reaction [32]. 250 *μ*L of TBA-TCA reagent was added to 250 *μ*L of colonic homogenate. The reagent comprises of 15% (w/v) of trichloroacetic acid (TCA); 0.375% (w/v) of 2-thiobarbituric acid (TBA); 15 mg of butylated hydroxytoluene (BHT); and 200 *μ*L of 0.25 N hydrochloric acid. The solution was kept in a sonicator for half an hour and gently heated on a magnetic stirrer for about 1 hr to assist the dissolution of TBA. After addition, these samples were heated on a water bath for about 40 min at 80°C. After heating, the samples were centrifuged at 10,000 rpm for 10 min at 4°C. The supernatant was transferred to a 96-well plate for measuring the absorbance at 432 nm. The concentrations of MDA were determined by constructing a standard plot by 1,1,3,3-tetramethoxypropane.

### 2.7. Nitrite Assay

During inflammation, macrophages and neutrophilic granulocytes of intestinal mucous membrane are activated and release large amounts of toxic NO [33], which would damage the intestinal mucous membrane or even react with superoxide anion (O_2_^•−^) and produce more active oxidizing substance called oxidized nitrous acid (OONO^−^). The cell membranes and organelles contain proteins and lipids which are oxidized by these oxidizing species and destruct the tissue in terms of free radical chain reaction so that the integrity of mucus membranes as a barrier is destroyed. In this assay, 100 *μ*L of sample (serum or colonic tissue homogenate) was taken and to this 100 *μ*L of Griess reagent was added in a 96-well plate. The absorbance was measured at 540 nm by placing the plate undisturbed in dark for 10 min. The concentrations were calculated with standard plot by using sulfanilamide as standard.

### 2.8. Statistics

The results were expressed as mean ± SEM of six readings. Statistical significance was calculated by analysis of variance (ANOVA) followed by posthoc Tukey's multiple comparison test by using Prism 5.03 Demo Version (GraphPad Software, Inc., La Jolla, CA, USA). *P* < 0.05 was considered to be significant.

## 3. Results

### 3.1. Body Weight

**Figure 1:**
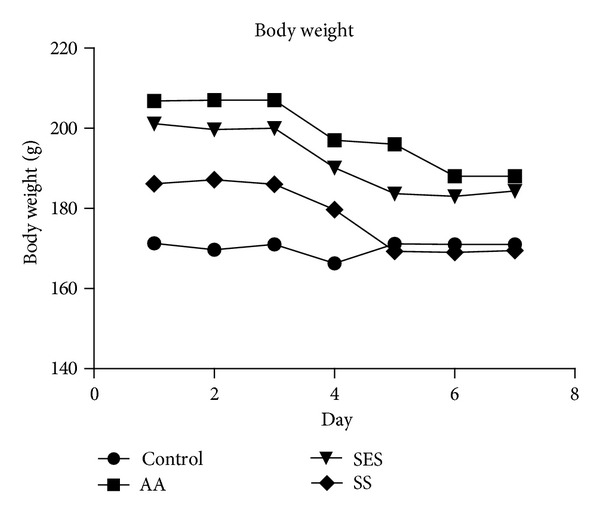
Effects of sesamol and sulfasalazine on the body weight of albino rats. ^a^ *P* < 0.05 as compared to positive control.

Figure 1 shows that the intracolonical administration of acetic acid caused the body weight to decrease from the 4th day onwards and continued until the 7th day, that is, the day of sacrifice. Compared to control there was a significant weight loss in AA and SES groups which were found to be 188.0 ± 5.0, 181.6 ± 7.95, and 184.4 ± 2.4 g, respectively, at *P* < 0.05, whereas there was no significant weight loss in SS group which was found to be 169.5 ± 2.32. It was also found that there was a significant difference between the test drug (SES) and standard drug (SS) at *P* < 0.05.

### 3.2. Colon Weight

**Figure 2:**
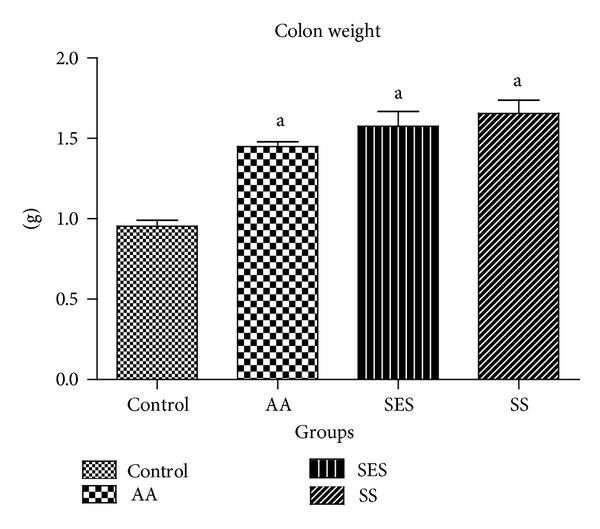
Effects of sesamol and sulfasalazine on colon weight of rats. ^a^ *P* < 0.05 as compared to positive control.

From Figure 2, it can be seen that weight of the colon increased significantly at *P* < 0.05 which was found to be 1.449 ± 0.029, 1.576 ± 0.091, and 1.655 ± 0.081 g in AA, SES, and SS treatment groups, but when compared to AA group, none of the treatments ameliorate this effect.

### 3.3. MPO Estimation

**Figure 3:**
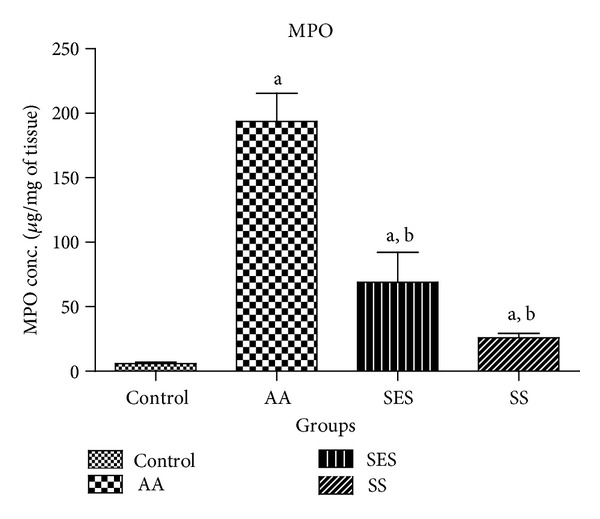
Effect of sesamol and sulfasalazine on the levels of MPO in tissue homogenates. ^a^ *P* < 0.05 as compared to positive control; ^b^ *P* < 0.05 as compared to AA group only.

Figure 3 depicts a significant rise in the levels of MPO at *P* < 0.05 in the AA group which was found to be 193.71 ± 21.86 *μ*g/mg of tissue. There was a significant decrease in the levels of MPO in drug treated groups (SES and SS) when compared to AA group at *P* < 0.05, which was found to be 68.95 ± 23.16 and 25.83 ± 3.33 *μ*g/mg of tissue, respectively. In between the standard and test drug treatment groups, no significant difference was observed.

### 3.4. Tissue Nitrite Estimation

**Figure 4:**
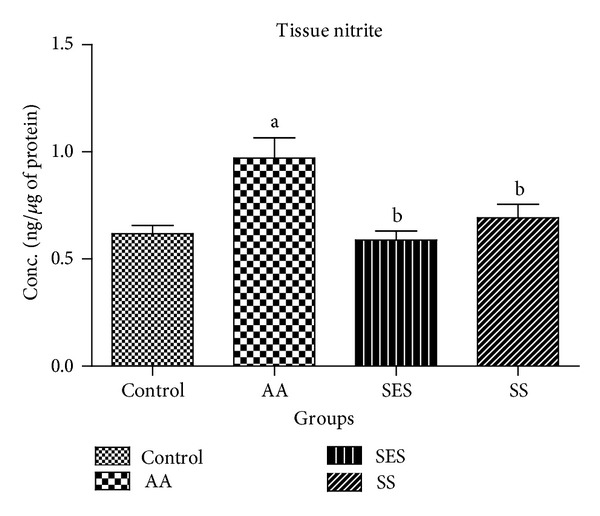
Effects of sesamol and sulfasalazine on the levels of tissue nitrite. ^a^ *P* < 0.05 as compared to positive control; ^b^ *P* < 0.05 as compared to AA group only.

It is evident from Figure 4 that when compared to the control group there was a significant rise in the levels of tissue nitrite at *P* < 0.05 in the AA group which was found to be 0.97 ± 0.094 ng/*μ*g of protein. Compared to AA group, both SES and SS treated groups showed a significant decrease in the levels of tissue nitrite which were found to be 0.58 ± 0.042, and 0.69 ± 0.064, respectively.

### 3.5. TBARS Estimation

**Figure 5:**
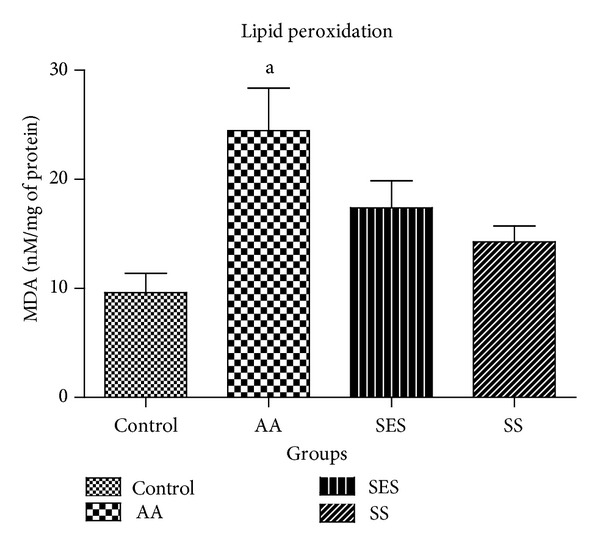
Effect of SES and SS on the concentration of MDA in the colonic tissue homogenates; ^a^ *P* < 0.05 as compared to positive control.

Figure 5 exhibits a significant rise in the levels of MDA in the AA group at *P* < 0.05 which is found to be 24.46 ± 3.89 nM/mg of protein. When compared to AA group, the levels of MDA were not significantly reduced at *P* < 0.05 in SES and SS treatment groups and were found to be 17.38 ± 2.468, and 14.25 ± 1.452 nM/mg of protein, respectively.

### 3.6. Estimation of Serum Nitrite

**Figure 6:**
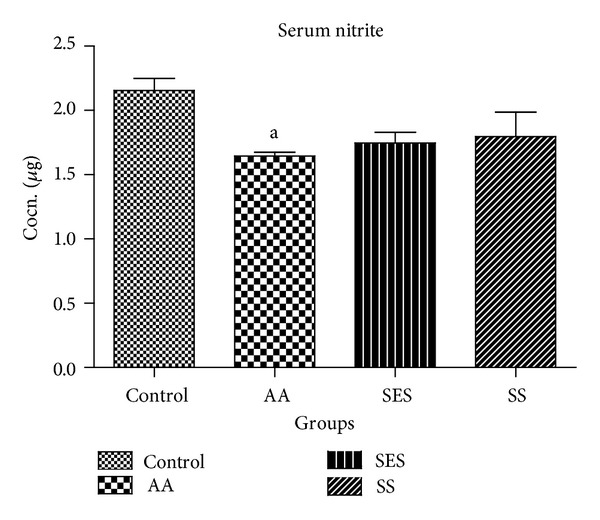
Effect of SES and SS on serum nitrite concentration; ^a^ *P* < 0.05 as compared to positive control.

Results were expressed as concentration of nitrite in *μ*g and also percentage decrease with respect to control in the serum. As shown in Figure 6, when compared to control only, AA induced group only showed a decrease in the levels of nitrite in the serum, whereas in the remaining groups the decrease was not found to be significant.

### 3.7. Histopathological Studies

**Figure 7:**
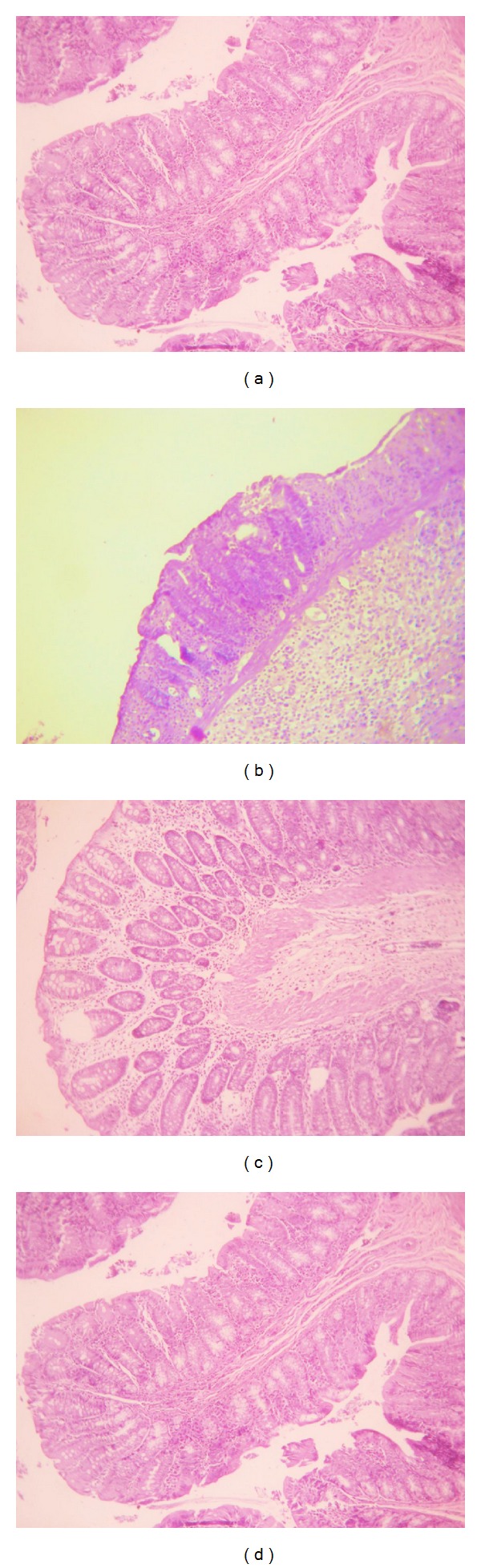
Histopathological studies; (a) normal control; (b) AA group; (c) SES treated; and (d) SS treated.

The normal histology of the colon was noted in the control (Figure 7(a)). In the AA treated group, colonic shrinkage of villi and mucosal layer was clearly seen (Figure 7(b)). Sesamol and sulfasalazine treated groups were similar to control (Figures 7(c) and 7(d), resp.) depicting normal crypts and few inflammatory cell infiltration.

## 4. Discussion

Inflammatory bowel disease is a disorder in which both autoimmune and immune mediated disorders are involved [34]. Of the two forms, especially in UC, an autoantigen named human tropomyosin isolated form 5 (hTM5) plays an important role in the activation of humoral and cellular mediated responses [35].

Modification of factors associated with IBD results in provision of relief to the patients. Apart from these factors, reactive oxygen species (ROS) also play an important role in the progression of the disease [36, 37]. Hence, we have selected sesamol (SES) which has a proved anti-inflammatory [24] and antioxidant activity [23] but its role in acetic acid-induced model of IBD has not been found out.

The mechanism by which AA induces colitis involves the entry of protonated form of acid into the epithelium where it dissociates to liberate protons causing intracellular acidification that might account for the epithelial injury [38].

In the present study, the actions of SES was assessed depending upon the gross (body weight and colon weight) and biochemical parameters (MPO, lipid per-oxidation and nitrite).

Weight loss is mainly due to abdominal pain and anorexia [39]. There was significant reduction in the body weight in the AA and SES treatment groups when compared to control which was ameliorated in the SS treatment group.

Weight of colon is raised due to the inflammation and also because of the increased activity of the fibroblasts leading to the overgrowth of muscularis mucosa [40]. All the groups showed significant rise in the weight of colon when compared with control.

Myeloperoxidase activity gives a quantitative measure of disease severity and a method of evaluating drug action in animal models of intestinal inflammation [31]. In our experiment, myeloperoxidase activity in the inflamed colon was determined. The drug SES was able to produce a reduction in the MPO activity, which can be considered as a manifestation of the anti-inflammatory activities of the test compound in the AA model.

In the present study, AA induced group showed a significant rise in the lipid peroxides which is indicative of oxidative stress [41]. The test drug was able to combat oxidative stress by reducing the colonic tissue contents of lipid peroxides but not in a significant manner.

Nitric oxide plays an important role in the pathogenesis of IBD [42, 43] and the levels of NO have been found to be raised in UC, CD, and toxic megacolon [44, 45]. NO levels in the colon of AA groups were found to be significantly raised when compared with normal control group which is in accordance with the earlier reports [46]. The treatment groups (SES and SS) showed a significant decrease in the levels of NO which indicated the mucosal protective activity of these compounds. This was further confirmed with histopathology findings. In contrast the levels of serum NO levels were found to be decreased in the AA group which is not correlating with the previously mentioned report due to unknown reason [46]. Sesame oil is shown to accelerate the healing activity in TNBS induced colitis model [27]. One of the constituents of roasted sesame oil, sesamol, proved that this is the main active constituent, because of its effective antioxidant [23], free radical scavenging properties [25] able to suppress MPO, TBARS, and NO. So, we might conclude that the drug, namely, SES showed a comparable activity with SS and also protected the mucosa from the harmful effects of acetic acid.

## Abbreviations

CD: Crohn's disease
SES: Sesamol
AA: Acetic acid
IBD: Inflammatory bowel disease
MPO: Myeloperoxidase
ROS: Reactive oxygen species
SS: Sulfasalazine
TBARS: Thiobarbituric acid reactive substrate
UC: Ulcerative colitis.
